# Embolization therapy for pelvic arteriovenous malformations: a systematic review

**DOI:** 10.1186/s42155-026-00667-x

**Published:** 2026-02-28

**Authors:** Chiara Brechbühl, Nicolas Diehm, Hanno Hoppe

**Affiliations:** 1 Microtherapy Center Bern, Lindenhofgruppe, Bremgartenstrasse 117, Bern, 3012 Switzerland; 2 Faculty of Medicine, University of Bern, Murtenstrasse 11, Bern, 3008 Switzerland; 3 Department of Diagnostic, Interventional and Pediatric Radiology (DIPR), Inselspital, Bern University Hospital, University of Bern, Rosenbühlgasse 27, Bern, 3010 Switzerland; 4 Vascular Institute Central Switzerland, Aarenaustrasse 2B, Aarau, 5000 Switzerland; 5 University of Applied Sciences Furtwangen, Jakob-Kienzle-Strasse 17, Villingen-Schwenningen, 78054 Germany; 6 Department of Health Sciences and Medicine, University of Lucerne, Frohburgstrasse 3, Lucerne, Postfach 4466, 6002 Switzerland

**Keywords:** Congenital pelvic arteriovenous malformations (AVMs), Embolization, Dominant outflow vein (DOV), Preferred Reporting Items for Systematic reviews and Meta-Analyses (PRISMA), Core Grading of Recommendations Assessment, Development and Evaluation (GRADE), PROSPERO International Prospective Register of Systematic Reviews

## Abstract

A pelvic arteriovenous malformation (AVM) is a rare congenital vascular anomaly that may cause relevant clinical symptoms. This systematic review synthesizes current evidence on diagnostic approaches, embolization techniques, and clinical outcomes for pelvic AVMs. A PRISMA (Preferred Reporting Items for Systematic reviews and Meta-Analyses)-compliant literature search identified 19 studies comprising 49 patients treated with embolization therapy. Venous approaches, including direct puncture of a dominant outflow vein (DOV), were most commonly performed. Coils were the primary embolic agent, frequently combined with adjunctive materials, reflecting substantial technical heterogeneity. Despite this variability, embolization achieved consistently high technical and clinical success rates with low complication rates over a mean follow-up of 21.1 months. Embolization therapy of DOV may be considered as first-line therapy for symptomatic pelvic AVMs, although future comparative studies are needed to guide standardized treatment strategies.

**Graphical Abstract:**

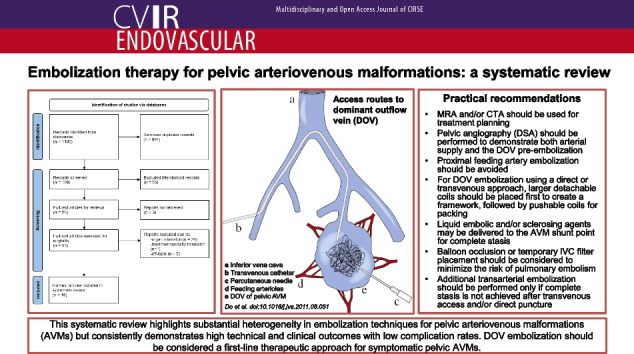

## Background

Pelvic arteriovenous malformations (AVMs) are rare vascular abnormalities characterized by direct, anomalous connections between arteries and veins that bypass normal capillary networks. Although AVMs are more commonly encountered in the brain, lungs, or kidneys, pelvic AVMs present unique diagnostic and therapeutic challenges because of their complex anatomy and diverse clinical manifestations, including pelvic pain, constipation, hematuria, menorrhagia, and hemorrhage [1].

**Fig. 1:**
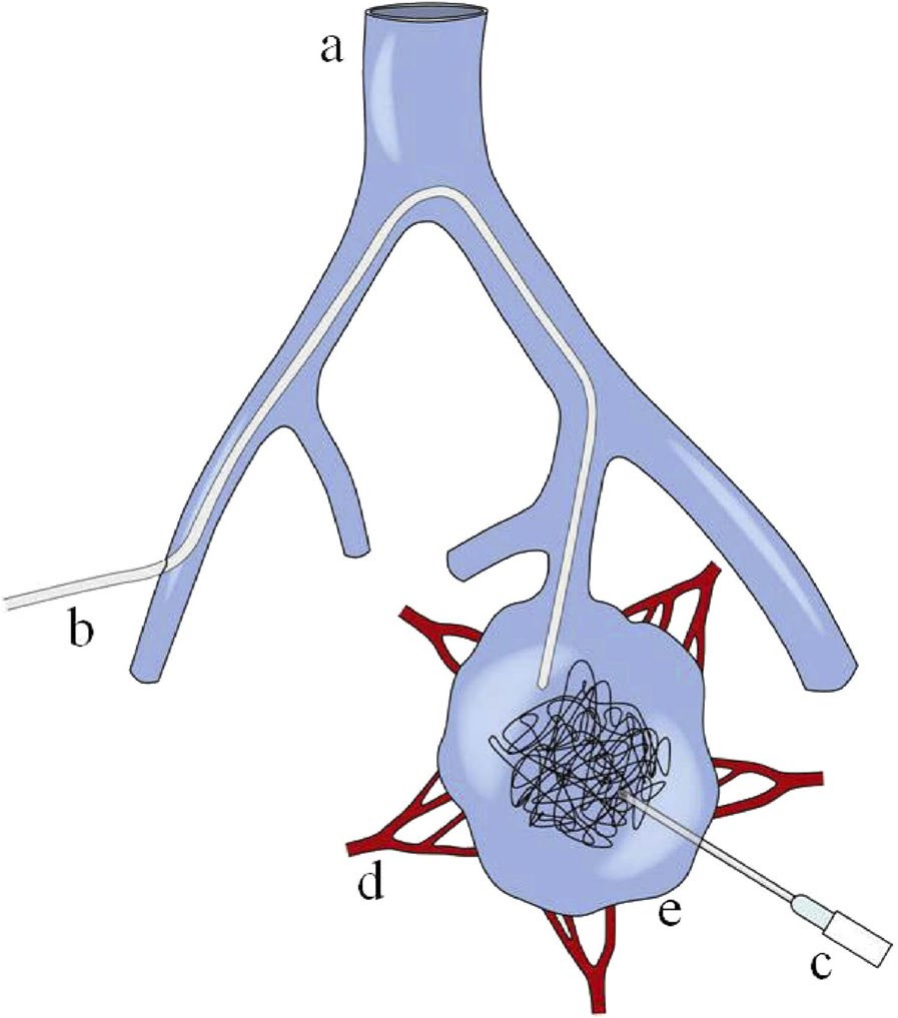
Schematic drawing of access routes to a dominant outflow vein (DOV) during the embolosclerotherapy of the pelvic AVM. **A** Inferior vena cava, **B** transvenous catheter, **C** percutaneous needle, **D** feeding arterioles, **E** DOV of a pelvic AVM. *From: Do YS, Kim YW, Park KB, Kim DI, Park HS, Cho SK, Shin SW, Park YJ. Endovascular treatment combined with emboloscleorotherapy for pelvic arteriovenous malformations. J Vasc Surg. 2012 Feb;55(2):465–71.*https://doi.org/10.1016/j.jvs.2011.08.051*. PMID: 22,051,867. Elsevier*

Congenital pelvic AVMs arise from embryological vascular malformations, whereas uterine AVMs are mainly considered to be acquired, often secondary to uterine trauma such as curettage or cesarean section. This study focuses on congenital pelvic AVMs, which generally exhibit a complex vascular architecture with multiple feeding arteries and draining veins, whereas acquired AVMs more often manifest as single fistulous communications [2]. Given their distinct anatomical and hemodynamic characteristics, pelvic AVMs are appropriately classified as a separate entity within Cho Type II AVMs [3]. Dysplastic arteries, usually originating from the internal iliac artery, form arteriovenous shunts with dominant outflow veins (DOVs) that typically drain into the internal iliac vein. The arteriovenous shunting of blood flow results in increased venous pressure, enhanced flow, and often marked vascular dilation within the venous system (Fig. 1) [4].

Pelvic AVMs frequently involve both the osseous and soft tissues of the pelvic wall. Doppler ultrasound serves as the initial diagnostic modality, often demonstrating turbulent, high-velocity flow. In addition, more specific cross-sectional imaging, such as computed tomography (CT) angiography or magnetic resonance (MR) angiography, should be performed to delineate lesion extent, surrounding structures, and feeding and draining vessels [5, 6]. A hallmark imaging feature of pelvic AVMs is rapid contrast enhancement of draining veins due to high-flow arterial shunting. Nevertheless, digital subtraction angiography (DSA) remains the gold standard, providing precise vascular localization essential for treatment planning [7].

In patients who are asymptomatic, conservative management with close observation and regular follow-up is considered safe and effective [8–10]. Intervention is typically reserved for patients with symptoms or complications, including bleeding or high-output cardiac failure. Surgical resection of pelvic AVMs is challenging because of the intricate vascular network, rapid reperfusion through collaterals, and the risk of complications, including bleeding or organ injury [11–14]. Accordingly, endovascular approaches, particularly embolization therapy, are preferred due to their minimally invasive nature associated with high technical and clinical success rates and low complication rates [15]. However, no consensus currently exists regarding the optimal embolization technique or choice of embolic agent [11].

The purpose of this systematic review was to assess the available evidence from studies on embolization of pelvic AVMs, focusing on procedural techniques, embolic materials, and clinical outcomes, to inform the best clinical practices and guide future research.

## Methods

To ensure consistency, transparency, and methodological rigor, a comprehensive and systematic literature review was conducted according to the PRISMA (Preferred Reporting Items for Systematic reviews and Meta-Analyses) protocol across five electronic databases (PubMed, Embase, Web of Science, Cochrane Library, Scopus, and Google Scholar) covering relevant records published from December 2022 to October 2025 to evaluate the current evidence regarding safety, efficacy, and clinical outcomes of embolization therapy for pelvic AVMs [16]. Keywords included “pelvic”, “pelvic AVM”, “pelvic arterio-venous malformation”, “pelvic AVM embolization” and “pelvic arterio-venous malformation embolization”. Boolean operators (AND, OR) were used to combine search terms. Advanced search strategies were employed to refine the study design and publication type, with a focus on peer-reviewed meta-analyses, case reports, and observational studies. Additionally, filters for publication date and language, limited to English, were applied.

Each full-text article was manually reviewed by two independent readers (CB and HH) to confirm eligibility. Selected studies were graded for evidence level based on adaptations from existing guidelines according to Table 1 [17, 18]. A standardized data extraction form, as depicted in Tables 2 and 3, was developed to collect relevant information from each included study by two independent reviewers.

**Table 1:** Levels of evidence adapted from the american society of plastic surgeons and Johns Hopkins nursing evidence-based practice: models and guidelines [18]

Level	Description
1	High quality prospective randomized controlled trials, cohort studies with adequate power, or systematic reviews of these studies
2	Lower quality prospective cohort, retrospective cohort studies, randomized controlled trials with untreated controls, or a systematic review/meta-analysis of these studies
3	Case–control studies or systematic review/meta-analysis of these studies
4	Case series, consensus statements, society guidelines, or practice guidelines
5	Expert opinion: case report, clinical example, or narrative reviews

**Table 2:** Standardized data extraction summarizing relevant information from each included study. Data with focus on level of evidence, GRADE score, and diagnostic criteria

Study	Year	Level of evidence	Grade score	Pat. no	Gender/age	Lead symptoms	Imaging	Side	Arteries	Veins	Vein size (mm)	AVM type
Kwon et al.	2025	4 (case series)	E1 moderate (suggest coil embolization)	1	F/49	pelvic pain	CT and/or MR	N/A	N/A	DOV	31	IIa
				2	F/30	asymptomatic	CT and/or MR	N/A	N/A	DOV	30	N/A
				3	M/49	pelvic pain	CT and/or MR	N/A	N/A	DOV	61	N/A
				4	M/62	asymptomatic	CT and/or MR	N/A	N/A	DOV	50	N/A
				5	M/33	asymptomatic	CT and/or MR	N/A	N/A	DOV	31	N/A
				6	F/43	asymptomatic	CT and/or MR	N/A	N/A	DOV	45	N/A
				7	M/23	pelvic pain	CT and/or MR	N/A	N/A	DOV	38	N/A
				8	M/62	pelvic pain	CT and/or MR	N/A	N/A	DOV	51	N/A
				9	M/42	thigh pain	CT and/or MR	N/A	N/A	DOV	28	N/A
				10	M/28	asymptomatic	CT and/or MR	N/A	N/A	DOV	15	N/A
				11	F/54	pelvic pain	CT and/or MR	N/A	N/A	DOV	59	N/A
				12	F/53	asymptomatic	CT and/or MR	N/A	N/A	DOV	27	N/A
				13	F/20	pelvic pain	CT and/or MR	N/A	N/A	DOV	23	N/A
Ku et al.	2025	5 (case report)	E4 very low	1	F/27	cardiac symptoms, dyspnea	CT	L	IIA	DOV	N/A	N/A
Amarneh et al.	2025	4 (case series)	E1 moderate (suggest US guidance for direct access)	1	M/18	gastrointestinal bleeding	CT, MR, US, DSA	bilateral	N/A	SMV	33	N/A
				2	M/3	gastrointestinal bleeding	CT, MR, US, DSA	R	N/A	IMV	12	N/A
				3	M/13	gastrointestinal bleeding	CT, MR, US, DSA	L	N/A	SMV	9	N/A
				4	F/25	portal hypertension	CT, MR, US, DSA	R	N/A	SMV	45	N/A
				5	M/58	asymptomatic	CT, MR, US, DSA	L	N/A	IIV	33	N/A
				6	F/31	cardiac symptoms	CT, MR, US, DSA	bilateral	N/A	IIV	42	N/A
				7	F/23	asymptomatic	CT, MR, US, DSA	R	N/A	DOV	9	N/A
				8	F/30	asymptomatic	CT, MR, US, DSA	bilateral	N/A	IIV	16	N/A
				9	M/23	asymptomatic	CT, MR, US, DSA	R	N/A	DOV	45	N/A
Inaguma et al.	2025	5 (case report)	E4 very low (suggest preop. embolization)	1	M/77	fatigue	CT, DSA	L	IIA	saphenous vein	N/A	N/A
Szmygin et al.	2025	4 (case series)	E1 moderate (suggest embolization is safer than surgery)	1	N/A	N/A	CT/MR, US, DSA	N/A	IMA	N/A	N/A	N/A
				2	N/A	N/A	CT/MR, US, DSA	N/A	IIA	N/A	N/A	N/A
				3	N/A	N/A	CT/MR, US, DSA	N/A	sacral artery	N/A	N/A	N/A
Viglione et al. 2024	2024	5 (case report)	E4 very low (suggest venous access)	1	M/42	pelvic pain	CT, US	bilateral	IIA, IMA	IIV	38	N/A
Sitharthan et al.	2024	5 (case report)	E4 very low (suggest preop. embolization)	1	M/82	urinary symptoms, enlarged prostate	CT, DSA	R	IIA	IIV	N/A	N/A
Izumi et al.	2024	5 (case report)	E4 very low (suggest embolization)	1	M/58	varices L lower extremity	CT, DSA	L	IIA	DOV	N/A	II
Gao et al.	2024	5 (case report)	E4 very low (suggest embolization)	1	F/17	gastrointestinal bleeding	CT, MR, DSA	R	IIA, IMA, sacral artery	N/A	N/A	N/A
Osuga et al.	2024	5 (case report)	E4 very low (suggest ethanol embolization via balloon)	1	M/40	gastrointestinal symptoms, headache	CT, DSA	R	IIA, inferior epigastric artery	DOV	N/A	II
Yang et al.	2024	5 (case report)	E4 very low (suggest embolization)	1	F/40	abdominal pain, lower back pain	N/A	L	uterine artery	DOV	52	N/A
Chan et al.	2023	5 (case report)	E4 very low (suggest embolization)	1	M/69	cardiac symptoms	CT, US, DSA	R	IIA, lumbar arteries	IIV	100	II
Nazari et al.	2023	5 (case report)	E4 very low (suggest embolization)	1	F/36	urinary symptoms (hematuria)	CT	bilateral	IIA	IIV, femoral vein	N/A	N/A
Huang et al.	2023	5 (case report)	E4 very low (suggest US for diagnosis)	1	M/30	abdominal pain, lumbar symptoms	CT, US	R	IIA	N/A	N/A	N/A
Ahmad et al.	2023	5 (case report)	E4 very low (suggest embolization with Squid)	1	M/29	N/A	CT, MR, DSA	L	IIA	N/A	N/A	N/A
Sagayanathan et al.	2023	5 (case report)	E4 very low (suggest embolization)	1	M/60	pelvic pain	CT, MR, US	R	IIA	N/A	N/A	N/A
Nguyen et al.	2023	5 (case report)	E4 very low (suggest embolization)	1	M/70	urinary symptoms, enlarged prostate	CT, US, DSA	R	IIA	N/A	N/A	N/A
Grill et al.	2023	4 (case series)	E4 very low (suggest embolization)	1	N/A	pelvic pain	MR	N/A	N/A	N/A	N/A	N/A
				2	N/A	pelvic pain	MR	N/A	N/A	N/A	N/A	N/A
				3	N/A	pelvic pain	MR	N/A	N/A	N/A	N/A	N/A
Kim et al.	2022	4 (case series)	E1 moderate (suggest preop. embolization)	1	F/52	cardiac symptoms, dypnea	CT	R	lumbar artery, ovarian artery, IMA	IIV	N/A	IIb
				2	F/49	vaginal bleeding	CT	R	IAA	IIV	N/A	IIb
				3	F/61	asymptomatic	CT	R	IAA	IIV	N/A	IIb
				4	F/58	abdominal pain	CT	R	IAA	IIV	N/A	IIb
				5	F/58	abdominal pain	CT	L	IAA	IIV	N/A	IIb
				6	F/32	asymptomatic	CT	R	IAA	IIV	N/A	IIb
				7	F/52	abdominal pain	CT	L	lumbar artery, ovarian artery, IMA	IIV	N/A	IIb

*Abbreviations*: *L* left, *R* right, *CT* computed tomography, *MR* magnetic resonance imaging, *N/A* not applicable, *F* female, *M* male, *DOV* dominant outflow vein, *IIA* internal iliac artery, *IIV* internal iliac vein, *IMA* inferior mesenteric artery, *E* evidence level

**Table 3:** Standardized data extraction summarizing relevant information from each included study. Data with focus on therapeutic criteria

Study	Year	Pat. no	Gender/age	Access	Embolic material	Technical success	Clinical success	Complications	Follow-up (months)
Kwon et al.	2025	1	F/49	venous	coils (54)	yes	yes	no	10.5
		2	F/30	direct	coils (19)	yes	yes	no	12.7
		3	M/49	venous	coils (59)	yes	yes	mild	N/A
		4	M/62	venous	coils (53)	yes	yes	no	23.5
		5	M/33	venous	coils (27)	yes	yes	no	N/A
		6	F/43	direct	coils (30)	yes	yes	no	21.8
		7	M/23	direct	coils (21)	yes	yes	no	N/A
		8	M/62	direct	coils (238)	yes	yes	no	9.1
		9	M/42	venous	coils (7)	yes	yes	no	24.3
		10	M/28	venous	coils (74)	yes	yes	no	39
		11	F/54	venous	coils (57)	yes	yes	no	4.5
		12	F/53	venous	coils (23)	yes	yes	no	14.2
		13	F/20	venous	coils (62)	yes	yes	no	1
Ku et al.	2025	1	F/27	arterial	N/A	N/A	N/A	N/A	N/A
Amarneh et al.	2025	1	M/18	direct	N/A	yes	N/A	minor venous access complication	N/A
		2	M/3	direct	N/A	yes	N/A	minor venous access complication	N/A
		3	M/13	direct	N/A	yes	N/A	minor venous access complication	N/A
		4	F/25	direct	coils	yes	N/A	hemoperitoneum	N/A
		5	M/58	direct	coils, glue	yes	N/A	minor venous access complication	N/A
		6	F/31	direct	coils	yes	N/A	minor venous access complication	N/A
		7	F/23	direct	coils, glue	yes	yes	puncture renal artery	N/A
		8	F/30	direct	coils, glue	yes	N/A	minor venous access complication	N/A
		9	M/23	direct	coils, glue	yes	N/A	minor venous access complication	N/A
Inaguma et al.	2025	1	M/77	arterial, direct	coils, glue	yes	yes	no	3 weeks
Szmygin et al.	2025	1	N/A	arterial	N/A	N/A	N/A	N/A	N/A
		2	N/A	arterial	N/A	N/A	N/A	N/A	N/A
		3	N/A	arterial	N/A	N/A	N/A	N/A	N/A
Viglione et al. 2024	2024	1	M/42	direct	coils, glue	yes	yes	no	N/A
Sitharthan et al.	2024	1	M/82	arterial	coils, plug	yes	yes	no	N/A
Izumi et al.	2024	1	M/58	arterial	coils, glue, ethanol	yes	yes	no	12
Gao et al.	2024	1	F/17	arterial	glue	yes	N/A	N/A	1
Osuga et al.	2024	1	M/40	arterial	coils, ethanol	yes	N/A	no	12
Yang et al.	2024	1	F/40	venous	coils, particles	yes	yes	fever	12
Chan et al.	2023	1	M/69	arterial, venous	coils, glue	yes	yes	mild postembolization syndrome	3
Nazari et al.	2023	1	F/36	arterial, venous	coils, gelfoam, ethanol, sclerosant, particles	N/A	N/A	hematuria	N/A
Huang et al.	2023	1	M/30	arterial	coils	no	no	no	19
Ahmad et al.	2023	1	M/29	arterial	Squid	partial (50%)	yes	no	24
Sagayanathan et al.	2023	1	M/60	arterial	Onyx	yes	yes	no	N/A
Nguyen et al.	2023	1	M/70	arterial	glue	yes	yes	no	2
Grill et al.	2023	1	N/A	N/A	N/A	N/A	N/A	N/A	N/A
		2	N/A	N/A	N/A	N/A	N/A	N/A	N/A
		3	N/A	N/A	N/A	N/A	N/A	N/A	N/A
Kim et al.	2022	1	F/52	arterial, venous	coils (84), ethanol	partial (> 80%)	N/A	N/A	186
		2	F/49	arterial, venous	coils (233), ethanol	yes	N/A	N/A	36
		3	F/61	venous	coils (69), ethanol	yes	NIA	N/A	24
		4	F/58	venous	coils (53), ethanol	yes	N/A	N/A	18
		5	F/58	venous	coils (183), ethanol	yes	N/A	N/A	18
		6	F/32	venous	coils (89)	yes	N/A	N/A	13
		7	F/52	venous	coils (266)	yes	N/A	N/A	6

*Abbreviations*: *L* left, *R* right, *CT* computed tomography, *MR* magnetic resonance imaging, *N/A* not applicable, *F* female, *M* male, *DOV* dominant outflow vein, *IIA* internal iliac artery, *IIV* internal iliac vein, *IMA* inferior mesenteric artery, *E* evidence level

Studies were graded according to their relevance or importance to the field using the best-practice GRADE score [19]. This score is implemented for the rating of evidence certainty using the GRADE system, which categorizes the reliability of evidence into four levels: “High, moderate, low, and very low”. This score is developed to help users understand how much they can trust the results of a study or guideline, with “high” indicating high confidence that the true effect is similar to the estimated effect and “very low” indicating very little confidence.

Exclusion criteria included primarily duplicates, recurrent articles by the same authors, abstracts, inaccessible full-text articles, unpublished data, article languages other than English, and reviews or case reports published before December 2022, because these had already been included in a previous review [11]. Furthermore, studies focusing on AVMs in anatomical locations other than the pelvis, notably uterine AVMs, were excluded. Moreover, records investigating solely treatment modalities other than embolization therapy (e.g., surgery or conservative therapy) and publications not consistent with the objectives and topic of our research, were ruled out.

The review protocol was registered in the PROSPERO database (International Prospective Register of Systematic Reviews) prior to the initiation of the study to avoid duplication and ensure accountability (CRD420251207131).

## Results

**Fig. 2:**
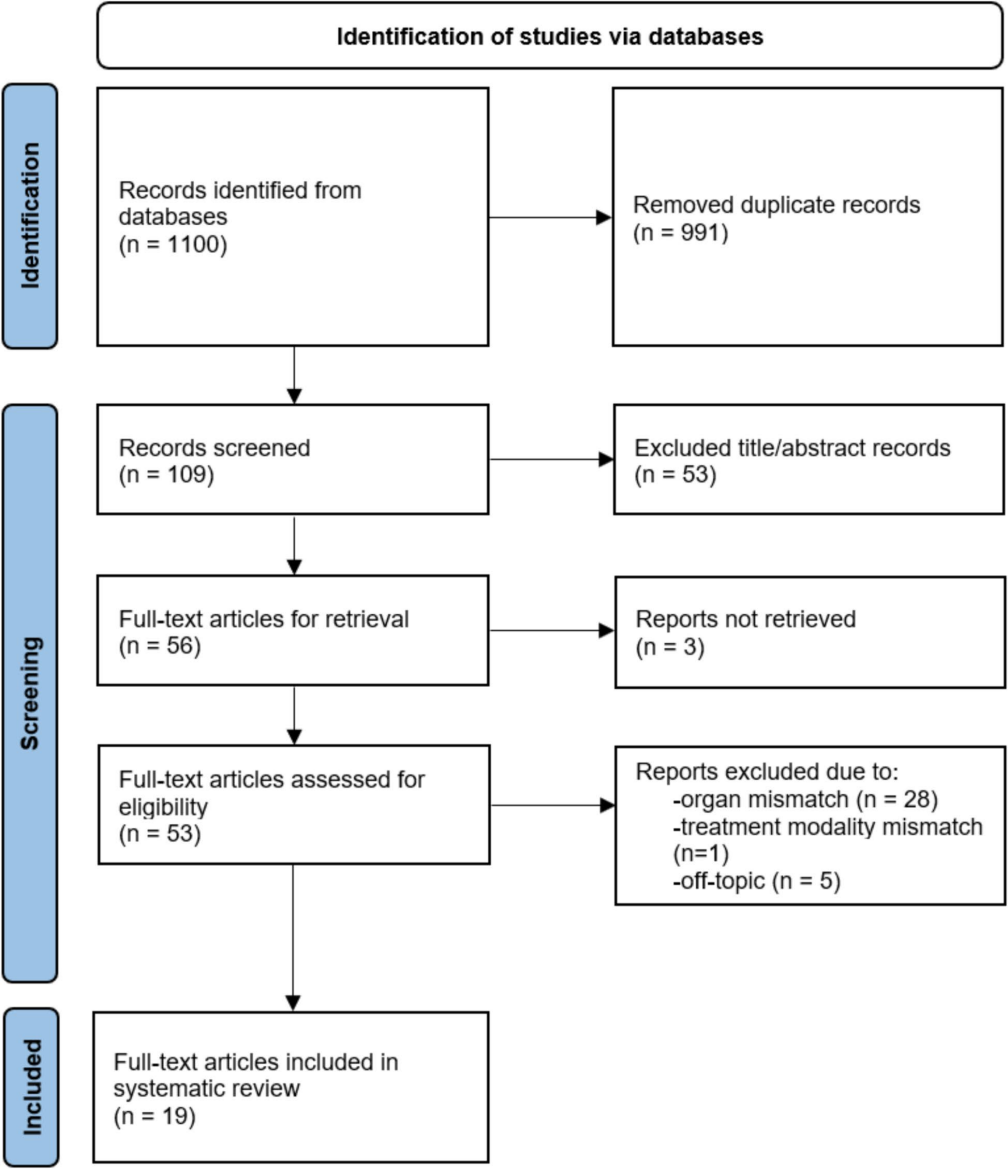
Preferred Reporting Items for Systematic reviews and Meta-Analyses (PRISMA) flow diagram demonstrating the study selection process, which consists of the following four stages: identification, screening, eligibility, and inclusion

The PRISMA flow diagram depicted in Fig. 2 elucidates the study selection process, which consists of the following four stages: identification, screening, eligibility, and inclusion:

1. *Identification*: A total of 1,100 records were initially identified from the six databases, all of which were imported into a reference management software (EndNote).
2. *Screening*: After exclusion of 991 duplicates and repeated articles, 109 unique titles and abstracts were screened independently by two reviewers to identify potentially relevant studies. Discrepancies were resolved by consensus.
3. *Eligibility*: Fifty-three full-text articles were retrieved and subsequently assessed for eligibility based on predefined exclusion criteria. The reasons for exclusion at each stage were documented.
4. *Inclusion*: Nineteen studies met all eligibility criteria and were included in the final systematic review.

A total of 49 patients who underwent endovascular embolization for congenital pelvic AVMs from nineteen studies authored by Kwon, Ku, Amarneh, Inaguma, Szmygin, Viglione, Sitharthan, Izumi, Gao, Osuga, Yang, Chan, Nazari, Huang, Ahmad, Sagayanathan, Nguyen, Grill, and Kim were included in this systematic review [11, 20–37]. Of these, 22 patients were male, 21 were female, and six patients were not specified in terms of sex. The average patient age was 43.5 years (range 3 to 82 years).

Clinically, the predominant presenting symptom was abdominal and/or pelvic pain, which was reported in 17 patients. Additional clinical manifestations, including gastrointestinal bleeding (*n* = 4), cardiac symptoms (*n* = 4), urinary symptoms (*n* = 3), thigh pain, gastrointestinal symptoms, and individual cases of fatigue, vaginal bleeding, and lower extremity varicose veins, were recorded. Twelve patients were asymptomatic. In four patients, symptoms were not specified.

Diagnostic imaging modalities across the cohort varied, reflecting heterogeneity in clinical practice. CT alone was performed in 10 patients, while three patients underwent magnetic resonance imaging (MR) only, and 13 patients received either CT or MR. A trimodality approach was used, where three patients were assessed with a combination of CT, MR and DSA or CT or MR, sonography and DSA (*n* = 3). Nine patients underwent comprehensive multimodality imaging comprising CT, MR, sonography and DSA. Moreover, one patient had CT, MR and sonography, whereas three patients received CT, DSA and sonography, and four patients underwent CT followed by DSA. CT and sonography were performed in two patients. In one patient, imaging modalities were not reported.

Pelvic AVMs were predominantly localized to the right side in 16 patients, whereas left-sided AVMs were identified in nine patients, and bilateral involvement was observed in five patients. In 19 cases, anatomical identification was not further specified.

Arterial feeders comprised the internal iliac artery in 19 patients. Other arteries included the inferior mesenteric artery (*n* = 5), lumbar arteries (*n* = 3), ovarian arteries (*n* = 2), sacral arteries (*n* = 2), obturator arteries (*n* = 1), inferior epigastric arteries (*n* = 1), and uterine arteries (*n* = 1). In 25 patients, arterial AVM feeders were not specified.

Venous drainage characterized by a DOV was specified in 19 patients. Venous drainage sites included the internal iliac vein (*n* = 14), superior mesenteric vein (*n* = 3), inferior mesenteric vein (*n* = 1), greater saphenous vein (*n* = 1), and concurrent right femoral vein in one patient. Venous drainage was not specified in 11 patients. The average diameter of these draining veins was 37 mm, with a range of 9 to 100 mm.

With respect to the AVM classification, seven patients presented with a type IIb lesion, one exhibited a type IIa lesion, three showed a type II lesion, and the majority (*n* = 38) were not further classified, highlighting a frequent lack of standardized reporting.

The average number of treatment sessions per patient was 2.4 (range of 1 to 13). The number of treatment sessions was not specified for 14 patients.

The access routes for endovascular AVM treatment were transvenous access in 15 patients, direct AVM puncture in 14 patients, transarterial access in 12 patients, both transarterial and transvenous access in four patients (one patient with covered endovascular reconstruction of the aortic bifurcation), both the arterial and direct approaches were used in one patient and were not further specified in three patients.

Embolic materials used were frequently coils (*n* = 18), coils in combination with glue (*n* = 7), coils with ethanol (*n* = 6), coils with glue and ethanol (*n* = 1), coils in combination with a vascular plug (*n* = 1), coils and particles (*n* = 1), glue alone (*n* = 2), Squid (*n* = 1), Onyx (*n* = 1), and coils in combination with gelfoam, particles, ethanol, and sclerosant (*n* = 1). The number of coils used per patient ranged from 7 to 266. These findings indicate high variability in embolic material selection. In 10 patients, embolic materials were not specified.

Technical success of endovascular treatment was reported positive in 38 patients, with technical failure in four patients and partial technical success in two patients. Technical outcome data were not reported for the remaining five patients. Clinical success, reflecting complete symptom improvement or lesion resolution, was recorded in 23 patients. Additionally, one patient experienced clinical failure, whereas clinical success was not reported in 25 patients.

Complication rates, including minor adverse events, were low. Minor adverse events, such as hematoma and postembolization syndrome with febrile response, occurred in 11 patients, and only one patient experienced iatrogenic right renal artery puncture due to the high complexity of the overall intervention. In contrast, 21 patients had no significant complications, while the complication status was not further outlined in 16 cases.

Moreover, wide variability across all the studies regarding follow-up duration, with an average interval of 21.1 months, ranging broadly from 0.8 to 186 months, complicates a structural and comprehensive assessment of both short- and long-term outcomes.

Among the 19 included studies, 14 were classified as level 5 evidence (case reports), and five were graded as level 4 evidence (case series). In accordance with the best-practice GRADE score, 15 studies were graded E4 (very low), while four studies were graded E1 (moderate).

## Discussion

Previous studies have reported favorable outcomes for embolization therapy in patients with pelvic AVMs [20, 22, 37]. In the present review, most patients were classified as having Cho Type II AVMs [3], characterized by a distinct DOV draining into the internal iliac vein and primarily supplied by dysplastic branches of the internal iliac artery. In these patients, embolization targeting the DOV is considered the recommended treatment strategy [1]. Consistently, 71% (14/49) of the patients in the included studies underwent embolization of the draining veins, either using a transvenous approach or direct puncture at the shunt point where the feeding arteries converge into the DOV, which is critical for achieving successful outcomes [20, 22, 23, 29–31, 37].

### Embolization therapy

The selection of embolic agents is key to treatment effectiveness. Coils were the most frequently used material, employed either alone or in combination with liquid embolics such as glue (n-butyl cyanoacrylate) or gelfoam and Onyx [31]. Emerging agents such as PHIL (precipitating hydrophobic injectable liquids) may enhance procedural safety and efficacy in complex cases [38]. However, no standardized protocols for embolic agent selection are currently available [11]. In the present study, coils were the preferred embolic material for treating pelvic AVMs in more than 87% (34/39) of patients. Recently, Kwon et al. reported the outcome of embolization therapy in 13 patients with Type IIa pelvic AVMs (DOV) using transvenous access (*n* = 10) or direct puncture (*n* = 4) [20]. In their study, DOV embolization was performed exclusively using coils. Their technical success rate was 92.9% (94% in the present study), and symptoms improved in all patients. Only one minor complication, namely a retroperitoneal hematoma that spontaneously resolved, occurred. Therefore, coil embolization of pelvic AVMs was demonstrated to be safe and effective within a median follow-up period of 13 months, ranging from 1 to 39 months. This method may be advantageous when liquid embolic agents pose additional risks or are contraindicated.

Liquid embolic agents, such as glue and Onyx, provide deep nidus penetration and durable occlusion (94% technical success) but demand high operator precision to avoid nontargeted embolization [39]. Improvements in microcatheter technology have further enhanced safety profiles [40]. Bioresorbable microspheres and agents such as PHIL contribute to tailoring embolization with controlled release and a reduced risk of ischemia. Huang et al. reported an 89% success rate without major complications, warranting larger trials [32, 41].

In the present study, the combination of coils and liquid embolic agents was used in 36% (14/39) of patients [11, 22, 23, 30, 31, 37]. When a combined approach using coils and liquid embolic agents is employed, coil packing should be performed first, followed by the administration of liquid embolic agents such as glue or Onyx. Larger detachable coils should be used to ensure a stable framework, followed by pushable coils for packing. Liquid embolic or sclerosing agents such as ethanol or polidocanol may subsequently be delivered directly into the shunt point to achieve complete stasis. This stepwise strategy improves controlled distribution of liquid embolic agent, promotes endothelial reaction, and minimizes the risk of nontargeted embolization [10]. Only if complete occlusion cannot be achieved via transvenous access or direct puncture alone, adjunctive transarterial embolization may be required to accomplish definitive AVM closure [30, 42]. Using this approach, Ghanaati et al. reported a 100% technical success rate with no recurrences across a 24-month median follow-up period, underscoring its utility despite higher technical demand [43].

While a diagnostic pelvic arteriogram is advised prior to DOV embolization, transarterial AVM embolization is generally not recommended since this approach typically does not facilitate enduring AVM obstruction because new feeding vessels develop and may necessitate multiple embolization treatments for clinical success [21, 24–28, 32–35]. However, transarterial embolization may be useful for reducing arterial inflow prior to DOV embolization to create more favorable conditions for positioning and polymerization of embolic agents [42].

Interestingly, Kim et al. performed transvenous embolization with coils and ethanol in seven patients with pelvic AVM, with a technical success rate of 86% [37]. However, no data on clinical success or complications have been reported. The median follow-up period was 43 months, ranging from 6 to 186 months (in this systematic review, the average follow-up period was 21.1 months [range 0.8 to 186 months]). Park and colleagues treated 84 patients with Type II pelvic AVMs with coils and ethanol and reported a clinical success rate of 64%, despite almost 90% of patients achieved full recovery or significant improvement [43].

Follow-up studies have consistently reported substantial symptom relief and improved quality of life after embolization. However, recurrence remains a concern, particularly in high-flow or incompletely occluded AVMs, with rates of up to 15% reported, highlighting the need for careful procedural planning, vigilant follow-up, and multidisciplinary management [24, 44].

### Complications

Amarneh et al. evaluated embolization therapy for pelvic AVMs using direct puncture and coils and glue as embolic agents in nine patients who underwent a total of 25 procedures [22]. Their technical success rate was 100%. However, no follow-up data were available. Complications reported in their study included seven minor access complications, one case of hemoperitoneum, and one case of inadvertent right renal artery puncture. Other complications include mild postembolization syndrome, including fever (*n* = 3) and hematuria (*n* = 1) [20, 29–31]. Hence, the complication rate of the present study, as reported, was 39% (13/33).

Previously, Bae et al. treated three female patients with pelvic AVM using ethanol embolization (sclerotherapy), which had high complication rates (up to 25%), including bladder necrosis and ovarian insufficiency [45]. This emphasizes the high risk of ethanol-related complications such as pulmonary vasospasm, cardiovascular collapse, nerve injury, and skin necrosis. Similarly, Mallios et al. reported a complication rate of almost 30%, including major complications such as pulmonary embolism and femoral paresis, when liquid embolics were used via a transarterial approach [46].

Compared with liquid embolic agents, coil embolization appears to have a superior safety profile, and liquid embolic agents may carry increased risks of serious complications, including tissue necrosis and nontargeted embolization [45, 46]. Compared with transvenous or direct puncture, transarterial approaches are associated with higher complication rates [43].

The aforementioned risk of nontargeted embolization may be reduced by the use of proximal balloon occlusion or the placement of an optional IVC filter [1, 28, 47–49]. However, this approach may be somewhat limited in case of large-caliber DOVs. In the present study, the average DOV diameter was 42 mm, and the maximum DOV diameter was 100 mm [30]. Of interest is the so-called “push-through” method, which uses a vascular plug in the DOV for protection in combination with a previously placed detachable-tip microcatheter for retrograde AVM embolization [50].

### Imaging

In the present study, predominantly sonography, CT-angiography, and/or MR-angiography were employed for pre-interventional imaging and treatment planning. Sonography, including color Doppler, is most frequently the initial examination used to demonstrate a mass with prominent periuterine vessels demonstrating intense color signals, aliasing, and potential reversal of flow [32]. These are indicators of turbulent high-velocity blood flow. A peak systolic velocity of less than 40 cm/s is associated with conservative therapy, whereas peak systolic velocities of more than 60 cm/s usually require timely treatment [51].

Cross-sectional imaging with CT and MR is advantageous for delineating AVMs, including adjacent anatomical structures and vessels. Most importantly, fast contrast uptake of dilated draining veins caused by arterial shunting is demonstrated on CT-angiography and, even more accurately, on MR-angiography [6, 52]. Although MR-angiography is proposed to be the imaging modality of choice, owing to its high spatial resolution, CT angiography may be superior for depicting small-sized arteriovenous shunt points where feeding arteries coalesce into the DOV. However, DSA remains the gold standard for pelvic AVM diagnosis and treatment planning and may be performed as an antecedent outpatient procedure or on the same day of treatment as an inpatient procedure [7].

### Limitations

The data of this systematic review demonstrate heterogeneity concerning clinical presentation, imaging strategies, and embolization techniques in pelvic AVM management, with predominantly low levels of evidence. Furthermore, the data reported in the studies reviewed appear to be inconsistent, and data on long-term efficacy, recurrence predictors, and the impact on quality of life are lacking.

### Practical recommendations

- MRA and/or CTA should be used for treatment planning.
- Pelvic angiography (DSA) should be performed within the treatment session to demonstrate both arterial supply and the DOV before embolization and to monitor treatment progress.
- Proximal feeding artery embolization should be avoided.
- For DOV embolization using a direct or transvenous approach, larger detachable coils should be placed first to create a framework, followed by the use of pushable coils for packing.
- Liquid embolic and/or sclerosing agents may be delivered to the AVM shunt point for complete stasis.
- Balloon occlusion or temporary inferior vena cava (IVC) filter placement should be considered to minimize the risk of pulmonary embolism.
- Additional transarterial embolization should be performed only if complete stasis is not achieved after using transvenous access and/or direct puncture.

### Outlook

Ongoing technological advancements in imaging, embolic materials, and artificial intelligence-supported planning are expected to further refine treatment. Multidisciplinary care and expanded research focusing on standardized protocols in terms of procedural techniques andembolic agent selection, long-term outcomes, and equitable access are essential to meet current challenges to ultimately establish evidence-based guidelines. Moreover, current angiographic and cross-sectional imaging limitations prompt the exploration of three-dimensional printing and virtual reality for preprocedural simulation, although expertise and costs currently limit widespread use. The incorporation of psychosocial support and patient-reported outcome measures in clinical practice warrants consideration. Furthermore, robotic-assisted embolization and artificial intelligence–based tools may hold promise and offer future benefits for enhancing procedural precision, treatment planning, and outcome prediction.

## Conclusion

The consistently high technical and clinical success rates, coupled with low complication rates, support embolization therapy, especially of DOV, as first-line treatment for complex pelvic arteriovenous malformations, offering durable outcomes and clear advantages in the majority of patients compared with surgical resection. However, the notable proportion of cases lacking detailed classification and comprehensive outcome reporting highlights the need for comparative research on embolization techniques, the establishment of standardized study protocols, and the development of evidence-based clinical guidelines supported by rigorous and robust data analysis in future investigations.

## Data Availability

Study data and materials are available.

## Abbreviations

AVM: Arteriovenous malformation
DOV: Dominant outflow vein
PRISMA: Preferred Reporting Items for Systematic reviews and Meta-Analyses
GRADE: Grading of Recommendations Assessment, Development and Evaluation
PROSPERO: International Prospective Register of Systematic Reviews
CT: Computed tomography
MR: Magnetic resonance
DSA: Digital subtraction angiography
PHIL: Precipitating hydrophobic injectable liquids
IVC: Inferior vena cava
L: Left
R: Right
N/A: Not applicable
F: Female
M: Male
IIA: Internal iliac artery
IIV: Internal iliac vein
IMA: Inferior mesenteric artery
E: Evidence level
